# Prediction of Frequency for Simulation of Asphalt Mix Fatigue Tests Using MARS and ANN

**DOI:** 10.1155/2014/515467

**Published:** 2014-02-04

**Authors:** Ali Reza Ghanizadeh, Mansour Fakhri

**Affiliations:** Department of Civil Engineering, K.N.Toosi University of Technology, No. 1346, Vali Asr Street, Mirdamad Intersection, Tehran 19967-15433, Iran

## Abstract

Fatigue life of asphalt mixes in laboratory tests is commonly determined by applying a sinusoidal or haversine waveform with specific frequency. The pavement structure and loading conditions affect the shape and the frequency of tensile response pulses at the bottom of asphalt layer. This paper introduces two methods for predicting the loading frequency in laboratory asphalt fatigue tests for better simulation of field conditions. Five thousand (5000) four-layered pavement sections were analyzed and stress and strain response pulses in both longitudinal and transverse directions was determined. After fitting the haversine function to the response pulses by the concept of equal-energy pulse, the effective length of the response pulses were determined. Two methods including Multivariate Adaptive Regression Splines (MARS) and Artificial Neural Network (ANN) methods were then employed to predict the effective length (i.e., frequency) of tensile stress and strain pulses in longitudinal and transverse directions based on haversine waveform. It is indicated that, under controlled stress and strain modes, both methods (MARS and ANN) are capable of predicting the frequency of loading in HMA fatigue tests with very good accuracy. The accuracy of ANN method is, however, more than MARS method. It is furthermore shown that the results of the present study can be generalized to sinusoidal waveform by a simple equation.

## 1. Introduction

Fatigue cracking, due to repeated traffic load, is among the most common mode of failures that flexible pavements experience. The fatigue life of asphalt mixes in laboratory tests is commonly determined by applying a dynamic load having sinusoidal or haversine waveform with a specific frequency. The frequency of horizontal tensile stress and strain pulses in both longitudinal and transverse directions depends on several factors such as vehicle speed, loading properties, environmental conditions, and pavement structure.

Several relationships were proposed to predict the duration of vertical stress pulse at different depth of asphalt layers [1–6]. Nevertheless, the knowledge on the frequency of stress and strain pulses in horizontal directions at the bottom of the asphalt layer is still poorly understood.

Brown (1973) derived an equation to calculate the loading time as a function of vehicle speed and depth beneath the pavement surface. The loading time was considered as the average of the pulse times of the stresses in the three directions as obtained from the elastic layered theory. The relationship between the loading time *t* (s), depth *d* (m), and vehicle speed *v* (km/h) was as follows [7]:

(1) $$\begin{array}{c} \log\left(t\right)=0.5d+0.2-0.94\log\left(V\right). \end{array}$$

When (1) is plotted for different speeds and thicknesses between 150 and 400 mm, it can be seen that the approximation *t* = 1/*V* (*V* = average speed in km/h) is a reasonable fit for the range of thicknesses studied [8].

In the development of the Mathematical Model of Pavement Performance (MMOPP), Ullidtz (2005) used the loading time corresponding to the middle depth of the asphalt layer. It was calculated based on the simplified assumption that the load at that depth is uniformly distributed over a circular area with the radius of *a* + *h*_1_/2, where *a* is the radius of the contact area and *h*_1_ is the thickness of the asphalt layer. Based on this assumption, the time of loading is defined as follows [5]:

(2) $$\begin{array}{c} t=\frac{\left(2a+h_{1}\right)}{V}, \end{array}$$

where *t* is the time of loading, a is the radius of the contact area, *h*_1_ is the thickness of the asphalt layer, and *V* is the vehicle speed. According to Ullidtz (2005), since no reductions are made for the influence of dual tires or for lateral distribution of the loads, the results should be on the conservative side [5].

Garcia and Thompson (2008) measured the durations of longitudinal and transverse tensile strain pulses in four sections tested with the accelerated pavement testing machine (ATLAS) [9]. They found a very strong relationship between the longitudinal and transverse strain pulse durations. In general, the transverse pulse durations were significantly, about three times, higher than those in the longitudinal direction [9].

Robbins and Timm (2009) used the instrumented sections of the National Center of Asphalt Technology (NCAT) to validate the procedure proposed by the Mechanistic Empirical Pavement Design Guide (MEPDG) to calculate the longitudinal pulse duration [10]. They developed a regression model for the duration of longitudinal strain pulse at the bottom of asphalt layer with three variables as follows [10]:

(3) $$\begin{array}{c} d=j\ln\left(h\right)+V^{k}+T^{l}+m, \end{array}$$

where *d* is the strain pulse duration; *h* is the thickness of asphalt layer; *V* is the vehicle speed, and *T* is the mid-depth temperature with *j*, *k*, *l*, and *m* as regression coefficients.

Hernandez (2010) performed an experimental testing program at the Accelerated Pavement Load Facility (APLF) of Ohio University on four pavement test sections [11]. He studied the influence of load, temperature, offset, and thickness of asphalt layer on the amplitude and duration of strain pulses at the bottom of the asphalt layer in both longitudinal and transverse directions. These experimental results showed that the load amplitude does not affect the longitudinal pulse duration [11]. The influence of the offset was furthermore dependent on the magnitude of the applied load. According to Hernandez (2010), the procedures recommended by MEPDG and Hu et al. (2010) [6] are not suitable to predict the magnitude of the longitudinal pulse duration, overestimating the desired variable by more than 2.5 times [11].

Restrepo-Velez (2011) evaluated the effect of several factors on the duration of tensile strains at the bottom of asphalt layer [12]. The pavement responses were measured on the perpetual section AC 664, of the WAY-30 project. Similar to the observation by Hernandez [11], Restrepo-Velez (2011) also concluded that the pulse durations were higher at lower values of speed and temperature. Additionally, Restrepo-Velez (2011) compared the observed responses with pavement responses predicted using the MEPDG method and the multilayer elastic analysis software, JULEA. Based on this comparison, MEPDG procedure led to an overprediction of the strain pulse durations of around 80% compared to those measured in the field [12]. It is worth nothing that, some studies have shown that the loading times calculated from the longitudinal strain gauges fall within the method suggested by MEPDG method [9, 13]. Based on the results of these studies there is no agreement in applicability of MEPDG method for prediction of loading times in longitudinal direction. This is due to the fact that the MEPDG method was originally developed to compute the loading time resulted from vertical stress, not horizontal responses.

According to the viscoelastic analysis of 112 flexible pavement sections, Fakhri et al. (2013) proposed regression equations for determining the duration of tensile stress and strain pulses at the bottom of asphalt layer in both longitudinal and transverse directions [14]. Proposed equations were developed based on haversine waveform by means of weighted nonlinear regression. Using this method of regression, the fitted haversine waveform only depends on three parameters including the speed of moving wheel, the thickness of asphalt layer, and asphalt layer temperature [14].

A recent study showed that several parameters such as thickness of different layers, ratio of resilient modulus for two succeeding layers, and contact radius of tire affect shape and frequency of longitudinal and transverse response pulses at the bottom of asphalt layer [15]. All of these parameters should be therefore considered to build up a comprehensive method for predicting loading time (i.e., frequency) of longitudinal and transverse response pulses. To the best of our knowledge, no general relationship or method has been proposed for determining of frequency of tensile horizontal stress and strain pulses at the bottom of asphalt layers. Existing relations are basically only applicable to vertical stress durations and limited number of equations which were developed based on field measurement of horizontal strain pulses cannot be used generally in other situations, where the pavement section and loading characteristics are very different from the desired sections.

The objective of this paper is to propose two methods for predicting the frequency of tensile stress and strain pulses at the bottom of asphalt layer in both longitudinal and transverse directions based on haversine and sinusoidal waveforms. By applying these methods, the frequency of loading in HMA fatigue laboratory tests such as four-points bending beam and indirect tensile (IDT) fatigue tests can be determined more realistically based on pavement design and loading characteristics. Results of this study can be used for more realistic simulation of dynamic loading of asphalt mix fatigue tests in both stress control mode and strain control mode.

## 2. Establishment of Dataset

### 2.1. Analysis of Pavement Sections

**Figure 1:**
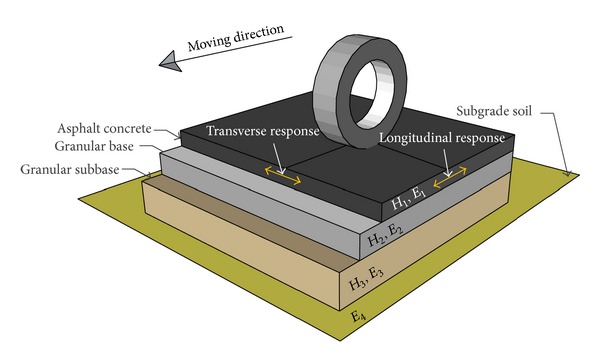
Characteristics of the Pavement structure and tensile strains.

In order to establish the dataset related to the normalized stress and strain pulses at the bottom of the asphalt layer, 5000 flexible pavement sections were analyzed using layered elastic theory (LET). The longitudinal and transverse stress and strain values were calculated at different radial distances from the center of the contact area. Moving load was assumed to be a single wheel having the contact pressure of 700 kPa. Since the contact pressure has no effect on the normalized shape and duration of response pulses, only the contact radius was considered as a variable. In each case, the pavement structure was considered as a four-layered system and all layers were treated as linear elastic. Interface of two succeeding layers was considered as fully-bounded. Pavement structure characteristics as well as tensile responses considered in this study are illustrated in Figure 1. The range of thickness of layers, the ratio of resilient modulus of each layer to the immediate succeeding layer, and the radius of the contact area are given in Table 1.

Each pavement section was analyzed using layered elastic analysis program, Non-PAS, which enables the analysis of a pavement system consisting of a maximum of ten linear or nonlinear elastic layers subjected to the maximum of ten circular contact loads. Detailed verification of Non-PAS program using Kenlayer program confirms that the Non-PAS program can accurately predict the pavement responses subjected to single and multiple loading [16]. Because of the limitation of the available pavement analysis programs such as Kenlayer and ELSYM 5 in respect to the number of response points, developing such a program was necessary. The developed code facilitates the calculation of responses (stresses, strains, and deflections) for an unlimited number of points in radial direction.

Stress and strain values in the longitudinal and transverse directions were computed at different radial distances with one centimeter interval, as long as the amount of tensile stress or tensile strain reduces to 1% of the maximum stress or strain, respectively.

**Figure 2:**
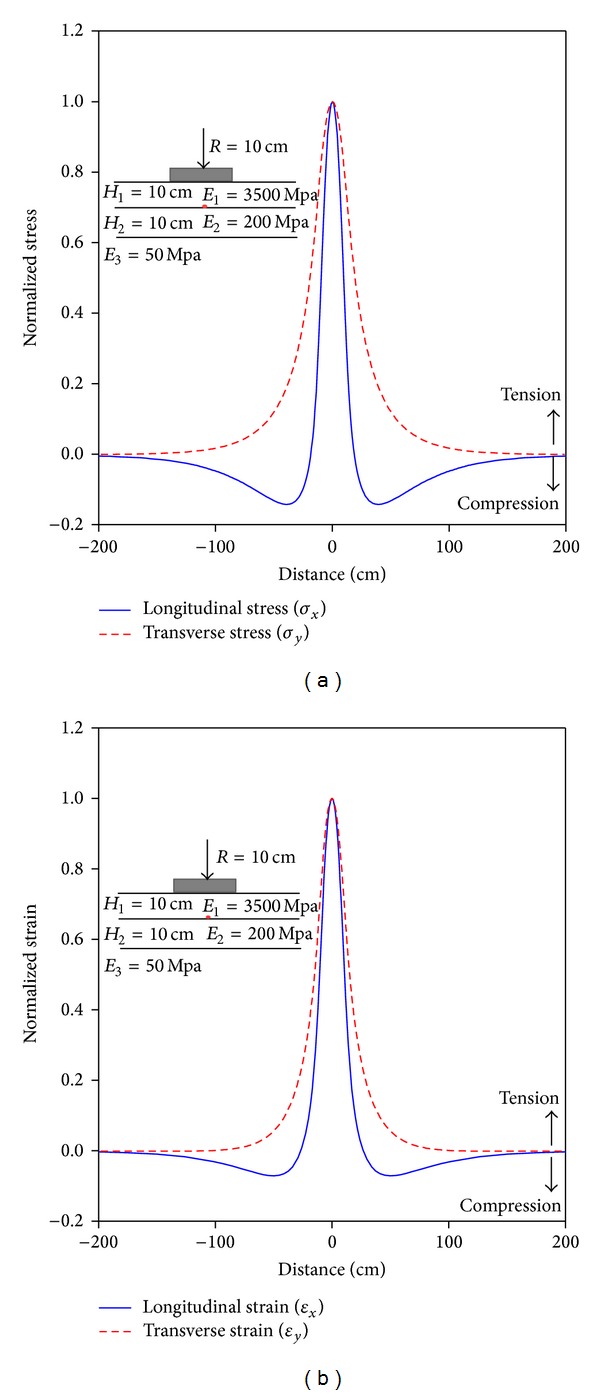
Normalized horizontal response versus distance for a sample pavement section.

The longitudinal and transverse stress and strain pulses at the bottom of HMA layer according to LET analysis are presented in Figure 2 for a typical pavement section.

As can be seen, the response pulse in longitudinal direction generally consists of two compression zones and one tension zone, while in case of transverse response pulse, the HMA layer commonly experiences tensile stress or strain. In general, the shape of strain pulses computed by NonPAS program was very similar to those measured in full-scale pavement tests [9–12]. In this research, with respect to prediction of loading frequency for simulation of asphalt fatigue damage, only the tension zone of longitudinal and transverse pulses was considered for modeling.

### 2.2. Fitting of the Haversine Function to the Analytical Response Pulse

Effect of loading waveform on the fatigue life of asphalt mixes can be explained by the energy put into the system per loading cycle [17]. It has been suggested that the energy is proportional to the area occupied by a load waveform in a stress (or strain) against time coordinate system [18]. On the other hand, full-scale test results showed that haversine function is a good representation of strain pulses in the longitudinal and transverse directions [9].

According to these observations, for each record of dataset (stress and stain pulses in both longitudinal and transverse directions at the bottom of asphalt layer for 5000 pavement sections), the haversine function was fitted to analytical response pulse such that the area under the haversine function was equal to the area under the analytical response pulse obtained by LET analysis. The normalized haversine function can be expressed as follows:

(4) $$\begin{array}{c} y\left(x\right)=\text{si}{\text{n}}^{2}\left(\frac{\pi }{2}+\frac{\pi x}{L_{\text{eff}}}\right), \end{array}$$

where *L*_eff_ is the effective length and *y*(*x*) is the normalized value of the pulse at distance of *x*. Since the analytical pulses have been determined in distance domain, duration of haversine pulse is also determined in distance domain which is called effective length (*L*_eff_). Given the speed of moving wheel, the duration of haversine pulse as well as loading frequency can be obtained using

(5) $$\begin{array}{c} d=\frac{L_{\text{eff}}}{27.78\;V}, \end{array}$$

(6) $$\begin{array}{c} f=\frac{1}{d}, \end{array}$$

where *d* is the duration in time domain in second, *L*_eff_ is the effective length based on fitted haversine waveform to analytical pulse in centimeter, *V* is the speed of moving wheel in km/h, and *f* is the frequency of haversine waveform in Hertz.

Before fitting the haversine function to response pulse, the response pulse was normalized by dividing all data points to the maximum value. The area under the normalized haversine function (*A*) can be obtained using the following integral:

(7) $$\begin{array}{c} A=\int_{-(L_{\text{eff}}/2)}^{+(L_{\text{eff}}/2)}\text{si}{\text{n}}^{2}\left(\frac{\pi }{2}+\frac{\pi x}{L_{\text{eff}}}\right)dx=\frac{L_{\text{eff}}}{2}. \end{array}$$

Given the area under the analytical response pulse (*A*_*p*_), the effective length (*L*_eff_) of fitted haversine function can be determined by *L*_eff_ = 2*A*_*p*_.

**Figure 3:**
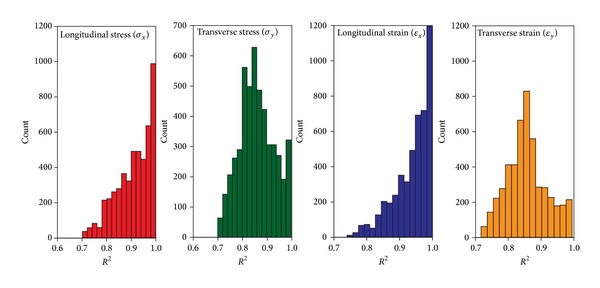
Frequency histogram of coefficient of determination (*R* ^2^) resulted from fitting haversine function.

As a criterion for goodness-of-fit, the coefficient of determination (*R* ^2^) between fitted haversine function and analytical response pulse was determined. The frequency histogram of coefficients of determination (*R* ^2^) is given in Figure 3. As evident, the maximum and minimum values of *R* ^2^ are 0.99 and 0.70, respectively. Figure 3 also indicates that the haversine function is fitted better to stress and strain pulses in longitudinal direction than transverse direction. This observation is in agreement with full-scale tests results [9].

Although the haversine waveform is more appropriate for representing the tensile responses at the bottom of asphalt layer (especially in transverse direction) and has been recommended by ASTM D7460, some standards such as AASHTO T321 and EN 12697-26 recommend the sinusoidal waveform to perform fatigue tests of asphalt mixes. In such cases, the equivalent effective length for sinusoidal wave shape can be computed easily by the following equation:

(8) $$\begin{array}{c} L_{\text{eff}}^{S}=\frac{\pi }{4}L_{\text{eff}}^{H}, \end{array}$$

where *L*_eff_^*S*^ is the effective length of a sinusoidal waveform which has the area equal to a haversine pulse with effective length of *L*_eff_^*H*^. In the next sections of this paper, the haversine waveform is considered for modeling of tensile response pulses at the bottom of asphalt layer. It is obvious that all results can be generalized to sinusoidal waveform by using (8).

### 2.3. Effective Length (*L*_eff_) of Horizontal Stress and Strain Pulse

The statistical data relating to computed effective length (*L*_eff_) for stress and strain pulses in both longitudinal and transverse directions is given in Table 2. As can be seen, in the case of both stress and strain pulses, the effective length in transverse direction is larger than that in longitudinal direction. The frequency of stress and strain pulses in both directions at two different speeds of 10 and 80 km/h are given in Table 3. Compared with the frequency of 5–10 Hz, which is commonly used in the asphalt mix laboratory test, the real frequency of loading may range from 0.30 to 77.5 Hz based on the vehicle speed, load specifications, and pavement design.

**Figure 4:**
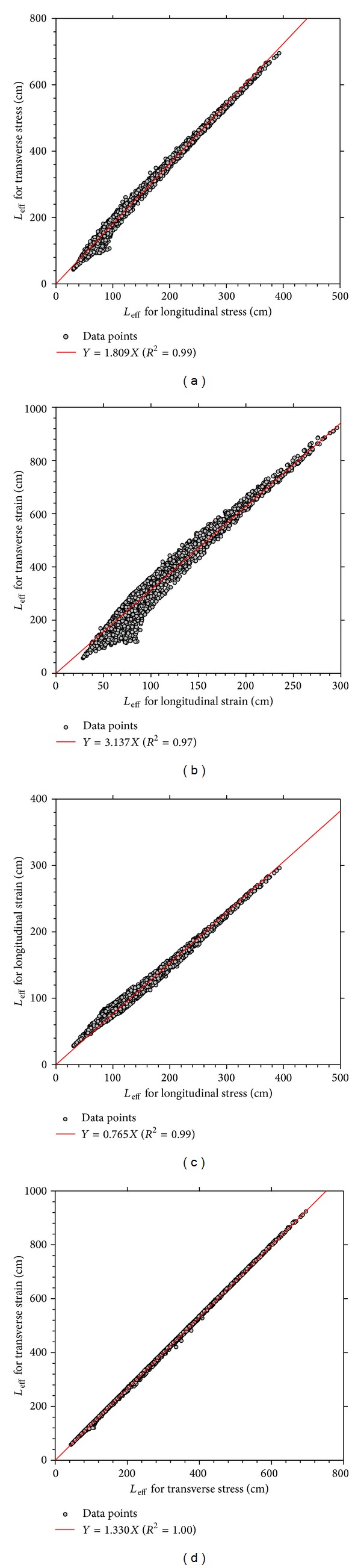
Relations between effective length of stress and strain pulses in longitudinal and transverse directions.

Relations between effective length of stress and strain pulses in longitudinal and transverse directions are illustrated in Figure 4. As can be seen, linear relationships exist between effective length of stress and strain pulses in both longitudinal and transverse directions. These relations are very useful and practical for computation of the effective length of different response pulses, where the effective length of one response pulse is known.

The ratio of effective length between transverse and longitudinal directions of strain pulse is about 3.14 (Figure 4(b)). This value is in agreement with the findings of previous researchers who observed that the duration of transverse tensile strain was almost three times of what was measured in longitudinal direction [9, 11].

## 3. Multivariate Adaptive Regression Splines (MARS)

### 3.1. Theory of MARS

The theory of Multivariate Adaptive Regression Splines (MARS) was developed by Friedman (1991) for solving regression-type problems [19]. The MARS technique has become particularly popular in the area of data mining because it is categorized into nonparametric regression procedures that make no assumption about the form of functional relationship (e.g., linear and logistic) between the dependent and predictor variables. Instead, useful models (i.e., models that yield accurate predictions) can be derived even in situations where the relationship between the predictors and the dependent variables is nonmonotone and difficult to approximate with parametric models [20].

MARS divides the whole space of input variable into various subregions. It defines a different mathematical equation for each subregion. The fundamental idea of MARS is to use the combination of the linear truncated basis functions to approximate the model. Thus, the functions of MARS consist of single spline functions or the product of two or more of the truncated power functions to allow for the interactions. MARS model can be written as follows:

(9) $$\begin{array}{c} f\left(X\right)=\beta_{0}+\sum_{n=1}^{N}\beta_{n}B_{n}\left(X\right), \end{array}$$

where *β*_0_ is the coefficient of the constant basis function *B*_0_(*X*) = 1, *B*_*n*_(*X*) is the *n*th basis function, which may be a single spline function or product of two or more, *B*_*n*_ is the coefficient of the basis function, and *N* is the number of basis functions in the model. Each basis function, *B*_*n*_(*X*), takes one of the three forms of the following: (1) a constant, (2), a hinge function (*x*_*i*_−*t*_*k*_)_+_ or (*x*_*i*_−*t*_*k*_)_−_ and (3) a product of two or more hinge functions. A product of two or more hinge functions can model interaction between two or more variables. The hinge functions have the following form:

(10) $$\begin{array}{c} {\left(x-t_{k}\right)}_{+}=\max\left(0,x-t_{k}\right)=\{\begin{array}{cc} x-t_{k}, & \text{if}\;x\geq t_{k}\\ 0, & \text{else} \end{array}\\ {\left(x-t_{k}\right)}_{-}=\max\left(0,t_{k}-x\right)=\{\begin{array}{cc} t_{k}-x, & \text{if}\;t_{k}\geq x\\ 0, & \text{else}, \end{array} \end{array}$$

where *t*_*k*_ is a constant, called the knot.

### 3.2. Learning in MARS

Model building in MARS is completed in two stages called the forward pass and the backward pass.

*The Forward Phase*. MARS begins with a model that just includes the intercept term and then adds basis function in pairs to the model successively. At each step it determines the pair of basis functions that provide the maximum reduction in sum-of-squares residual error. The two basis functions in the pair are identical except the case that a different side of a mirrored hinge function is used for each function. Each new basis function composed of a term already in the model is multiplied by a new hinge function. A hinge function is defined by a variable and a knot. Hence, to add a new basis function, MARS must search over all combinations of existing terms (parent terms), all variables (to select one for the new basis function), and all values of each variable (for the knot of the new hinge function). The forward phase is executed until one of the following conditions occurs: change in residual error is smaller than threshold or the maximum number of terms is reached. These parameters are specified by the user beforehand. Due to the nature of hinge functions, this search can be done more rapidly by using fast least-squares update technique or by using a heuristic method that reduces the number of parent terms considered at each step [19].

*The Backward Pass*. The forward pass usually builds an overfitted model. So, a backward deletion phase is engaged to build a model with better generalization ability. In this phase the model is pruned by removing one least effective to find the best submodel. Model subsets are compared using the generalized cross-validation criterion (GCV) which is defined as follows:

(11) $$\begin{array}{c} \text{GCV}=\frac{\left(1/N\right)\sum_{i=1}^{N}{\left[y_{i}-\hat{f}\left(X_{i}\right)\right]}^{2}}{{\left[1-(d\cdot M)/N\right]}^{2}}, \end{array}$$

where *M* is the number of basis functions in the model, $\hat{f}$ denotes the fitted values of the current MARS model, *N* denotes the number of data points and *d* is the penalizing parameter. The numerator is the common residual sum of squares, which is penalized by the denominator, which accounts for the increasing variance in the case of increasing model complexity. The penalizing parameter *d* can be chosen arbitrarily. A conventional value is *d* = 4. A smaller *d* generates a larger model with more basis functions; a larger *d* creates a smaller model with less basis functions [21].

At the end of the backward phase, from those “best” models of each size, a model with lowest GCV value is selected as the final one. One of the backward phase's benefits over the forward phase is that at any step it can select any term to delete, whereas the forward phase, at each step, can only see the next pair of terms. The forward phase adds terms in pairs; however, the backward pass typically discards one side of the pair. Thus, terms are often not seen in pairs in the final model [19].

### 3.3. Prediction of *L*_eff_ by Means of MARS

For developing some equations to predict the effective length (*L*_eff_) of stress and strain pulses at the bottom of asphalt layer in both longitudinal and transverse directions, on the basis of MARS method, STATISTICA program was employed. The degree of interactions between predictors was assumed to be 3, since higher values had no effect on improving the model. Maximum number of basis function and penalizing parameter *d* were set to 25 and 4, respectively. The following equations have been developed for prediction of effective length of stress and strain pulses at the bottom of asphalt layer.

Stress pulse in longitudinal direction (*σ*_*x*_):

(12) Leff=17.84649+4.68246×max⁡(0,H1−5)+0.031017×max⁡(0,H1−5)×max⁡(0,E1E2−39)−0.07433×max⁡(0,H1−5)×max⁡(0,39−E1E2)+0.24011×max⁡(0,H1−5)×max⁡(0,E2E3−4)−0.48707×max⁡(0,H1−5)×max⁡(0,4−E2E3)+0.35755×max⁡(0,H1−5)×max⁡(0,E3E4−30)−0.69655×max⁡(0,H1−5)×max⁡(0,30−E3E4)+3.21134×max⁡(0,R−10)+0.72980×max⁡(0,42−H2)+0.02613×max⁡(0,R−10)×max⁡(0,E1E2−26)−0.07210×max⁡(0,R−10)×max⁡(0,26−E1E2)+0.00223×max⁡(0,R−10)×max⁡(0,E1E2−26)×max⁡(0,E2E3−3)−0.01429×max⁡(0,R−10)  ×max⁡(0,E1E2−26)×max⁡(0,3−E2E3)+0.00806×max⁡(0,R−10)×max⁡(0,E1E2−26)×max⁡(0,E3E4−9)−0.00459×max⁡(0,R−10)×max⁡(0,E1E2−26)×max⁡(0,9−E3E4)−0.00671×max⁡(0,H1−5)  ×max⁡(0,E2E3−6)×max⁡(0,E3E4−30)−0.03664×max⁡(0,H1−5)×max⁡(0,6−E2E3)×max⁡(0,E3E4−30)−0.00108×max⁡(0,H1−5)×max⁡(0,H2−33)×max⁡(0,E3E4−30)−0.00193×max⁡(0,H1−5)×max⁡(0,33−H2)×max⁡(0,E3E4−30)−0.00375×max⁡(0,H1−5)×max⁡(0,H3−26)×max⁡(0,E3E4−30)+0.00186×max⁡(0,H1−5)×max⁡(0,26−H3)×max⁡(0,E3E4−30).

Stress pulse in transverse direction (*σ*_*y*_):

(13) Leff=50.82904+8.07127×max⁡(0,H1−5)+0.39907×max⁡(0,E1E2−39)−1.90639×max⁡(0,39−E1E2)+0.45301×max⁡(0,H1−5)×max⁡(0,E2E3−4)−0.90799×max⁡(0,H1−5)×max⁡(0,4−E2E3)+  0.36940×max⁡(0,H1−5)×max⁡(0,E3E4−3)−1.45047×max⁡(0,H1−5)×max⁡(0,3−E3E4)+4.76974×max⁡(0,R−10)+0.04838×max⁡(0,H1−5)×max⁡(0,E1E2−28)−0.12620×max⁡(0,H1−5)×max⁡(0,28−E1E2)−1.47860×max⁡(0,H2−36)+0.97541×max⁡(0,36−H2)−5.84342×max⁡(0,6−E2E3)+0.23175×max⁡(0,39−E1E2)×max⁡(0,E2E3−10)+0.37279×max⁡(0,39−E1E2)×max⁡(0,10−E2E3)+0.00324×max⁡(0,R−10)×max⁡(0,E3E4−3)−0.02905×max⁡(0,R−10)×max⁡(0,E1E2−28)×max⁡(0,E3E4−3)+0.003381×max⁡(0,R−10)×max⁡(0,28−E1E2)×max⁡(0,E3E4−3)−0.25111×max⁡(0,R−10)×max⁡(0,E2E3−3)×max⁡(0,E3E4−3)+  0.03116×max⁡(0,R−10)×max⁡(0,3−E2E3)×max⁡(0,E3E4−3).

Strain pulse in longitudinal direction (*ε*_*x*_):

(14) Leff=12.99845+3.31521×max⁡(0,H1−5)+0.02133×max⁡(0,H1−5)×max⁡(0,E1E2−41)−0.05544×max⁡(0,H1−5)×max⁡(0,41−E1E2)+2.91609×max⁡(0,R−10)+0.13670×max⁡(0,H1−5)×max⁡(0,E2E3−4)−0.41009×max⁡(0,H1−5)×max⁡(0,4−E2E3)+0.25174×max⁡(0,H1−5)×max⁡(0,E3E4−3)−0.45133×max⁡(0,H1−5)×max⁡(0,3−E3E4)−1.28586×max⁡(0,H2−47)+0.71949×max⁡(0,47−H2)−0.00315×max⁡(0,H1−5)×max⁡(0,41−E1E2)×max⁡(0,E2E3−7)+0.00739×max⁡(0,H1−5)×max⁡(0,41−E1E2)×max⁡(0,7−E2E3)−0.00993×max⁡(0,H1−5)×max⁡(0,E2E3−8)×max⁡(0,E3E4−3)−0.02422×max⁡(0,H1−5)×max⁡(0,8−E2E3)×max⁡(0,E3E4−3)+0.00139×max⁡(0,H1−5)×max⁡(0,E1E2−26)×max⁡(0,E3E4−3)−0.00618×max⁡(0,H1−5)×max⁡(0,26−E1E2)×max⁡(0,E3E4−3)+0.07548×max⁡(0,47−H2)×max⁡(0,E2E3−7)−0.06523×max⁡(0,47−H2)×max⁡(0,7−E2E3)−0.00235×max⁡(0,H1−5)×max⁡(0,H3−25)×max⁡(0,E3E4−3)+0.00384×max⁡(0,H1−5)×max⁡(0,25−H3)×max⁡(0,E3E4−3)+0.07573×max⁡(0,E1E2−26)−0.48934×max⁡(0,26−E1E2).

Strain pulse in longitudinal direction (*ε*_*y*_):

(15) Leff=52.60950+10.55170×max⁡(0,H1−5)+0.47535×max⁡(0,E1E2−39)−1.12822×max⁡(0,39−E1E2)+0.72181×max⁡(0,H1−5)×max⁡(0,E2E3−4)−1.07866×max⁡(0,H1−5)×max⁡(0,4−E2E3)+0.72353×max⁡(0,H1−5)×max⁡(0,E3E4−3)−1.82082×max⁡(0,H1−5)×max⁡(0,3−E3E4)+7.40323×max⁡(0,R−10)+0.05451×max⁡(0,H1−5)×max⁡(0,E1E2−28)−0.13696×max⁡(0,H1−5)×max⁡(0,28−E1E2)−1.81207×max⁡(0,H2−36)+1.49244×max⁡(0,36−H2)+0.86372×max⁡(0,R−10)×max⁡(0,E2E3−3)−1.44292×max⁡(0,R−10)×max⁡(0,3−E2E3)−0.02151×max⁡(0,R−10)×max⁡(0,53−E1E2)×max⁡(0,E2E3−3)+0.25010×max⁡(0,R−10)×max⁡(0,E2E3−3)×max⁡(0,E3E4−9)−0.10420×max⁡(0,R−10)×max⁡(0,E2E3−3)×max⁡(0,9−E3E4)+0.00364×max⁡(0,H1−5)×max⁡(0,E1E2−2.7)×max⁡(0,E3E4−3)−0.01644×max⁡(0,H1−5)×max⁡(0,2.7−E1E2)×max⁡(0,E3E4−3)−0.11419×max⁡(0,H1−5)×max⁡(0,E2E3−6)×max⁡(0,E3E4−3)−0.06901×max⁡(0,H1−5)×max⁡(0,6−E2E3)×max⁡(0,E3E4−3),

where *H*_1_ is thickness of asphalt layer (cm), *H*_2_ is thickness of base layer (cm), *H*_3_ is thickness of subbase layer (cm), *E*_1_/*E*_2_ is the ratio of asphalt resilient modulus to base resilient modulus, *E*_2_/*E*_3_ is the ratio of base resilient modulus to subbase resilient modulus, *E*_3_/*E*_4_ is the ratio of subbase resilient modulus to subgrade resilient modulus.

Regression statistics for (12) to (15) are given in Table 4.

**Figure 5:**
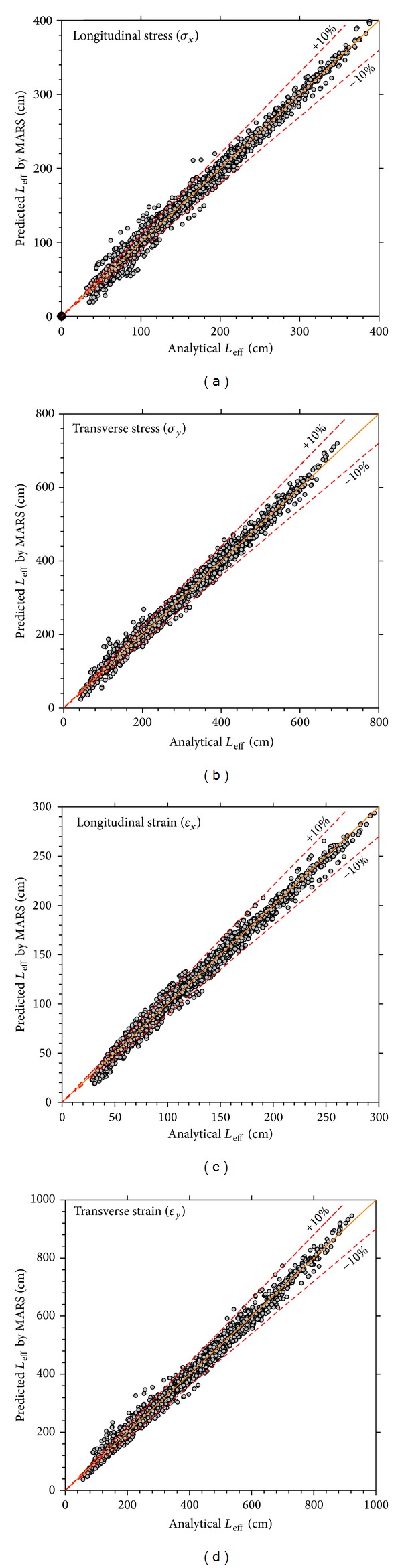
Capability of MARS method for predicting effective length (*L*_eff_).

Capability of (12) to (15) to predict the effective length of tensile stress and strain pulses in both longitudinal and transverse directions at the bottom of asphalt layer is illustrated in Figure 5. As evident, these equations give good accuracy to predict effective length. For all pulses, the error percentage decreases with increasing the effective length. It can be therefore concluded that for high values of effective length the proposed equations have sufficient accuracy; however, for low values of effective length (pavements with thick asphalt layer), proposed equations are of limited accuracy.

## 4. Artificial Neural Network (ANN)

### 4.1. Theory of ANN

**Figure 6:**
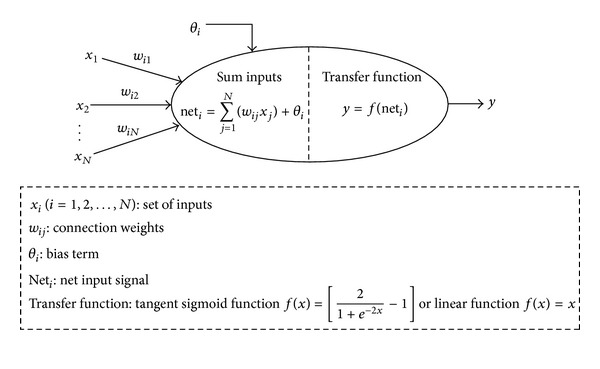
Summation and transfer functions of a typical artificial neuron.

In order to predict the effective length accurately, the Artificial Neural Network (ANN) method was employed. The ANN approach is a computer methodology which attempts to simulate some important features of the human nervous system; in other words, the ability to solve problems by applying the information gained from the past experiences to new problems or case scenarios. Analogous to a human brain, an ANN uses many simple computational elements, named artificial neurons, connected by variable weights [22]. A typical artificial neuron is illustrated in Figure 6. A neural network can be trained to perform a particular function by adjusting the values of the connections (weights) between the elements. Neural networks are trained so that a particular input leads to a specific target output. The network is adjusted based on a comparison of the output and the target until the network output matches the target. Typically many such input/target output pairs are used to train a network.

**Figure 7:**
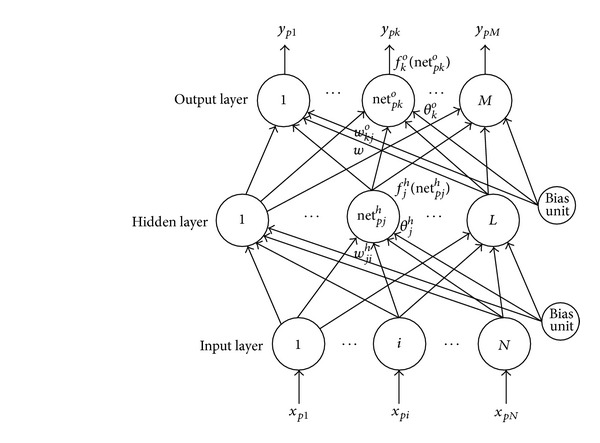
A three layer feed-forward backpropagation network architecture.

A feed-forward backpropagation neural network (Figure 7) is a kind of ANN which is useful in addressing problems requiring recognition of complex patterns and performing nontrivial mapping function [23].

The training of a feed forward neural network using a backpropagation algorithm involves the following two phases [24, 25].

(i) Forward Phase. During this phase, the free parameters of the network are fixed, and the input signal is propagated through the network layer by layer. The forward phase ends with the computation of an error signal using the following:
  (16) $$\begin{array}{c} e_{i}=d_{i}-y_{i}, \end{array}$$
  where *d*_*i*_ is the desired response and *y*_*i*_ is the actual output produced by the network in response to the input *x*_*i*_.
(ii) Backward Phase. During this second phase, the error signal *e* is propagated through the network in the backward direction, hence, the name of the algorithm. It is during this phase that adjustments are applied to the free parameters of the network so as to minimize the error *e* in a statistical sense.

### 4.2. Prediction of *L*_eff_ by Means of ANN

Several implementations of the backpropagation algorithm are possible. In the present study, the Levenberg-Marquardt algorithm was adopted due to its efficiency in training networks. This implementation is readily available in Matlab software within its neural network toolbox. The testing (30%), cross validating (10%), and training (60%) sets for ANN training procedure were selected randomly from the established dataset. Several networks with one hidden layer and different numbers of neurons in each hidden layer (5 to 20) were explored to determine the optimal architecture of BPN. The optimal architecture of ANN with the minimum possible size and acceptable accuracy was found to be 7-15-3 (15 hidden neurons) architecture. Hyperbolic tangent sigmoid and linear transfer functions were used for the hidden layer and output layer, respectively. More details to implement the proposed ANN for prediction of the effective length of different tensile responses at the bottom of asphalt layer are given in Appendix.

**Figure 8:**
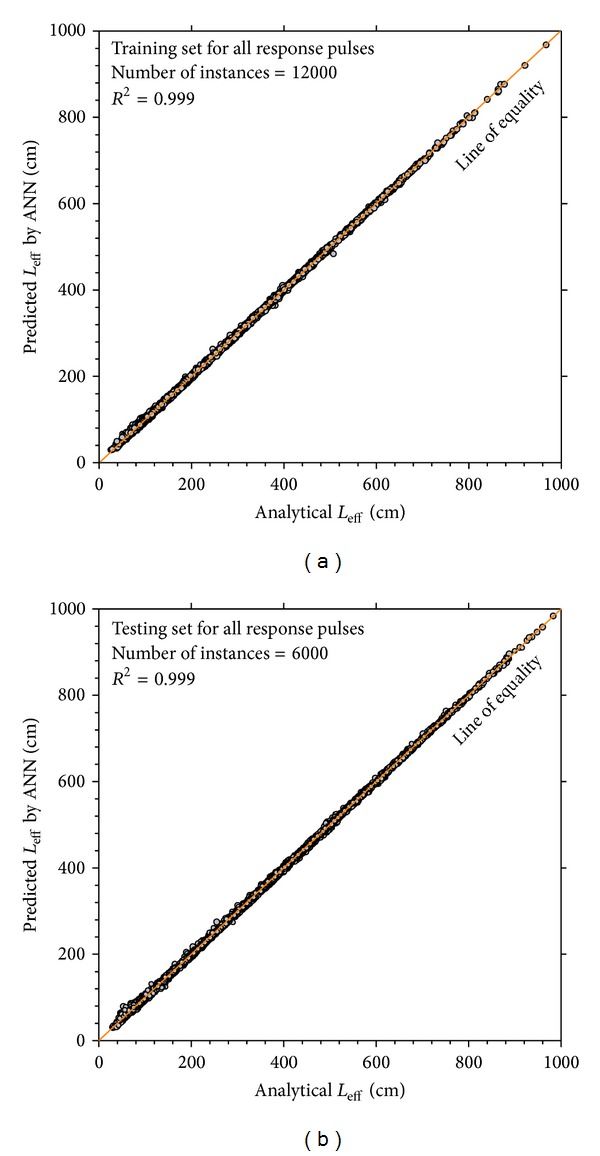
Performance of ANN for predicting effective length (*L*_eff_).

**Figure 9:**
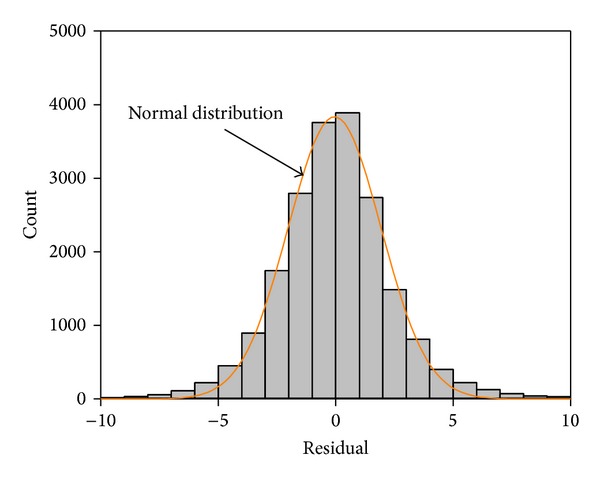
Frequency histogram of residuals resulted from ANN.

The performance of ANN modeling to predict the effective length (*L*_eff_) of longitudinal and transverse stress and strain pulses related to training and testing sets is demonstrated in Figure 8. The frequency histogram of ANN output residuals is presented in Figure 9 and is compared to normal distribution.

**Figure 10:**
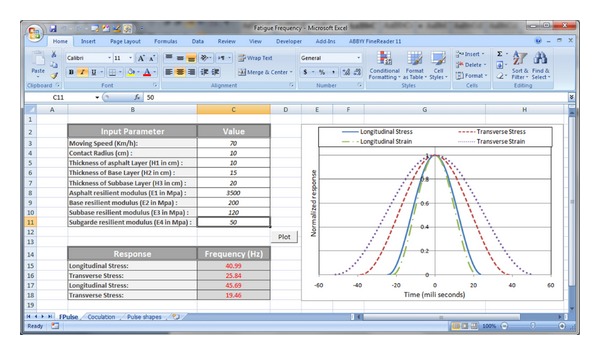
User interface of program for predicting loading frequency.

For prediction of frequency of different responses at the bottom of asphalt layer based on ANN, a program was developed using the macrocapability of Microsoft Excel. This program enables user to determine the frequency of stress and strain pulses at the bottom of asphalt layer with respect to input parameters (moving speed, thickness of layers, resilient modulus of different layers, and contact radius of moving load). User interface of this program is represented in Figure 10.

### 4.3. Parametric Analysis

A standard pavement section composed of a HMA layer, an aggregate base, an aggregate subbase, and subgrade soil was assumed to investigate the effect of various parameters on the effective length of the tensile response pulses. These controlling parameters include the radius of contact area, thickness of different layers, and ratio between the modules of each layer and the immediate succeeding layer below. The thickness and the resilient modulus of different layers are represented in Table 5. The standard contact radius for parametric analysis was considered to be 10 cm.

**Figure 11:**
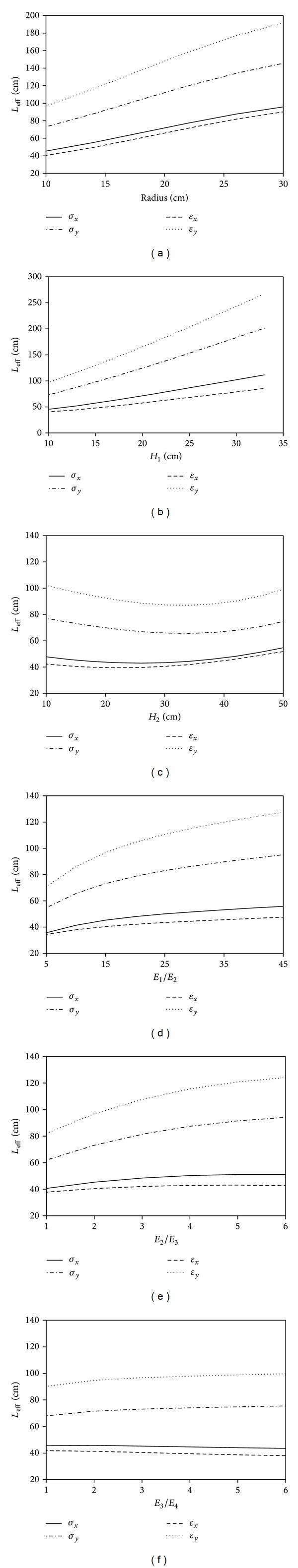
Effect of different parameters on effective length of tensile response pulses.

In order to study the effect of different parameters on the effective length of tensile stress and strain pulses, the ANN method was employed. Using ANN, the effective length of the pulse was computed by changing the desired parameter. The results of the parametric analysis are illustrated in Figure 11. The effect of subbase thickness (*H*_3_) is not shown, because, similar to *H*_2_, it has a very slight effect on effective length.

As can be seen in Figure 11, some parameters including the ratio of resilient modulus of subbase to subgrade (*E*_3_/*E*_4_) and the thickness of base and subbase layers have a slight effect on the effective length of response pulses, especially in longitudinal direction. The most prominent factors that affect the effective length of tensile response pulses are as follows:

- contact radius of wheel,
- thickness of asphalt layer,
- the ratio of resilient modulus of asphalt layer to base layer,
- the ratio of resilient modulus of base to subbase layer.

All of these factors have a direct effect on the effective length of tensile response pulses at the bottom of asphalt layer. The effective length of stress and strain pulses in both longitudinal and transverse directions increase with the increase of these parameters.

It is worth nothing that the effect of temperatures is similar to the reciprocal of *E*_1_/*E*_2_ ratio. In fact when the temperature increases, the *E*_1_/*E*_2_ ratio decreases and vice versa. Full-scale tests results show that the duration of tensile response pulses increases with decreasing the asphalt layer temperature which is in agreement with the effect of *E*_1_/*E*_2_ ratio on effective length [10–12].

## 5. Conclusions

The following conclusions can be drawn from this study.

1. Haversine function was fitted to stress and strain pulses in longitudinal direction better than transverse direction.
2. Response pulse in longitudinal direction generally consists of two compression zones and one tension zone, while in case of transverse response pulse, the HMA layer commonly experiences tensile stress or strain. The strain pulses computed by LET are very similar to those observed in full-scale pavement tests.
3. There are strong correlations between the effective length of stress and strain pulses in both longitudinal and transverse directions. Using these relations and measuring/computing the effective length for one of the stress or strain pulses, the effective length for other responses can be obtained easily.
4. MARS approach predicts the effective length with a good accuracy when the effective length is large (e.g., wide-base tires or pavement sections with thick asphalt layer). A more accurate method is therefore needed when the effective length is small.
5. A feed-forward backpropagation neural network with architecture of 7-15-4 accurately predicts the effective length (*L*_eff_) of tensile stress and strain pulses in both longitudinal and transverse directions at the bottom of the asphalt layer.
6. Based on ANN, an Excel-based computing code was developed to predict the frequency of different responses at the bottom of the asphalt layer.
7. The most prominent factors that affect the effective length of tensile response pulses are the contact radius of wheel, the thickness of asphalt layer, the ratio of resilient modulus of asphalt layer to the base layer, and the ratio of resilient modulus of base to sub-base layer. The effective length of tensile response pulses in both longitudinal and transverse directions increases with the increase of these factors.

## Figures and Tables

**Table 1:** Ranges of analytical dataset.

Statistical parameter	Radius	*H*_1_	*H*_2_	*H*_3_	*E*_1_/*E*_2_	*E*_2_/*E*_3_	*E*_3_/*E*_4_
Minimum	10.00	5.00	10.00	10.00	5.00	1.00	1.00
Maximum	30.00	40.00	50.00	50.00	100.00	10.00	10.00
Mean	19.88	22.59	30.05	29.74	52.13	5.49	5.48
Mode	11.00	29.00	43.00	50.00	20.00	1.00	1.00
Median	20.00	23.00	30.00	30.00	52.00	6.00	5.00
Standard deviation	6.07	10.36	11.89	11.95	27.60	2.84	2.88

*H*_*i*_: thickness of *i*th layer in centimeter.

*E*_*i*_/*E*_*j*_: ratio of *i*th layer modulus to *j*th layer modulus.

Radius of contact area is in centimeter.

**Table 2:** Statistical data relating to effective length (*L*_eff_) of tensile response pulses.

Statistic	Stress pulses		Strain pulses	
	*σ*_*x*_	*σ*_*y*_	*ε*_*x*_	*ε*_*y*_
Min., cm	30.74	43.74	28.67	57.50
Max., cm	392.66	694.63	295.92	922.08
Mean, cm	156.17	282.45	121.07	375.52
Mode, cm	60.19	254.30	237.20	735.47
Median, cm	145.72	269.22	111.95	357.64
Standard deviation, cm	73.89	134.15	53.16	178.90

**Table 3:** Range of computed frequencies (Hz) assuming two different speeds.

Speed, km/h	10		80		10		80	
Statistics	*σ*_*x*_	*σ*_*y*_	*σ*_*x*_	*σ*_*y*_	*ε*_*x*_	*ε*_*y*_	*ε*_*x*_	*ε*_*y*_
Max.	9.0	6.5	72.3	50.8	9.7	4.8	77.5	38.6
Min.	0.7	0.4	5.7	3.2	0.9	0.3	7.5	2.4
Mean	1.8	1.0	14.2	7.9	2.3	0.7	18.4	5.9

**Table 4:** MARS regression statistics.

Response	Mean (observed)	SD (observed)	Mean (predicted)	SD (predicted)	Mean (residual)	SD (residual)	*R* ^2^	GCV
*σ*_*x*_	156.4103	73.7415	156.4103	73.3139	0.0000	7.9300	0.988	64.52
*σ*_*y*_	283.1745	133.5965	283.1745	132.9735	0.0000	12.8876	0.991	169.24
*ε*_*x*_	121.1816	53.1068	121.1816	52.8136	0.0000	5.5725	0.989	31.90
*ε*_*y*_	376.5493	178.0933	376.5493	177.1838	0.0000	17.9760	0.989	331.57

SD: standard deviation.

**Table 5:** The standard pavement section for parametric analysis.

*H*_1_(cm)	*H*_2_(cm)	*H*_3_(cm)	*E*_1_/*E*_2_	*E*_2_/*E*_3_	*E*_3_/*E*_4_
10	15	20	15	2	3

**Table 6:** Weight matrix of hidden layer (*W* ^*h*^)^*t*^_15×7_.

0.24056638	0.52180021	−0.08192893	0.02059694	0.27453521	−2.15797251	0.16841500
0.14152848	−0.83464978	0.12392608	0.02935285	0.16551902	0.22003344	0.18289943
0.29412997	0.37355134	−0.13930790	−0.06150401	0.29807662	0.11364017	−2.01880910
−0.10907694	0.45073205	−0.07290811	−0.11726552	−0.04816373	0.52349298	−0.12122008
−0.56102272	0.25020881	0.18042916	0.08662090	0.03791313	0.00963579	−0.04494278
−1.20391196	2.49023600	−1.18313570	−0.53611691	2.05716497	−2.01891789	1.58568172
0.04309756	0.45406603	−0.22674448	−0.07681359	0.03332427	0.20049821	0.29780929
0.28555679	−0.55316048	0.28744294	0.11453127	−0.50806732	−0.29575540	−0.10511421
−0.00467866	−0.46119549	0.16664234	0.06328881	−0.17277587	−0.22346863	−0.12622049
−0.04098276	−1.22152659	0.63119917	0.10577187	−0.59785759	−0.14715576	0.27344356
−0.72460658	0.98059866	−0.00946927	−0.00763101	0.56841275	0.47432854	0.42334442
0.00739616	0.19834485	−0.00695040	−0.00229041	0.17336683	0.09259456	0.08924832
0.16358940	−0.33227832	0.24788596	0.11224513	−0.58502546	−0.28035227	−0.10703562
0.12400955	0.20304430	0.09051104	−0.01267492	−2.21543491	0.11016067	0.16626881
0.05108061	1.09864936	−0.56182159	−0.10324686	0.53970892	0.18221514	−0.22157991

**Table 7:** Weight matrix of output layer (*W* ^*o*^)_15×4_.

−0.30111544	−0.31546319	−0.24939048	−0.30986842
−0.64704052	−0.73747399	−0.61956350	−0.74215198
−0.46625917	−0.55965630	−0.41951552	−0.55530329
0.61036557	0.55902914	0.61154085	0.56048484
−0.37299472	−0.29700295	−0.48360558	−0.29897197
0.01711208	0.01797442	0.01231933	0.01817974
1.20349568	1.12822954	1.09308434	1.12441006
−0.88776052	−0.76191322	−1.03085965	−0.77482237
3.46074792	3.18511221	3.32861330	3.17908780
0.31898041	0.90058623	−0.18541501	0.86128651
−0.28043854	−0.32078608	−0.26632566	−0.33308883
4.35600143	4.33534570	4.28254176	4.34864238
1.08157418	0.98638475	1.19385865	0.98936233
−3.01939611	−2.79134501	−2.69827499	−3.00674084
0.76785271	1.36287794	0.18770446	1.31743751

**Table 8:** Bias vector of hidden layer (*θ* ^*h*^).

−2.98061282
−1.43752227
−3.28639956
0.51238963
0.34781783
1.86026187
0.33527476
0.83759704
−0.52129274
−0.41618793
−1.18229383
−0.33680531
0.90575109
−3.85056664
0.46806618

**Table 9:** Bias vector of output layer (*θ* ^*o*^).

−2.67535023
−2.76969939
−2.18693474
−2.97130086

## Appendix

### Weights and Biases of Artificial Neural Network (ANN)

This appendix is assigned to input vector, output vector, weight factors, and bias factors of the backpropagation network which was discussed in Section 4. The optimum architecture of backpropagation network is 7-15-4 with sigmoid transfer function in hidden layer and linear transfer function in output layer. The order of normalized predictors in the input vector is as follows:

(A.1) $$\begin{array}{c} I={\left\{R,H_{1},H_{2},H_{3},\frac{E_{1}}{E_{2}},\frac{E_{2}}{E_{3}},\frac{E_{3}}{E_{4}}\right\}}_{1\times 7}. \end{array}$$

The order of normalized output parameters in the output vector is as follows:

(A.2) $$\begin{array}{c} O={\left\{L_{\text{eff}}^{\sigma x},L_{\text{eff}}^{\sigma y},L_{\text{eff}}^{\varepsilon x},L_{\text{eff}}^{\varepsilon y}\right\}}_{1\times 4}. \end{array}$$

Before simulating of network, inputs and outputs should be normalized based on the following relation:

(A.3) $$\begin{array}{c} Q_{n}=2\frac{\left(\mathrm{Max}-\mathrm{Min}\right)}{\left(Q-\mathrm{Min}\right)}-1, \end{array}$$

where *Q*_*n*_ is normalized value of parameter *Q*, *Max*⁡ is maximum observed value for parameter *Q*, *Min*⁡ is minimum observed value for parameter *Q*. Maximum and minimum values of input and output parameters are given in Tables 1 and 2. Weight matrixes for hidden and output layers are given in Tables 6 and 7, respectively.

Bias vector for hidden and output layer is given in Tables 8 and 9, respectively.
